# Five-Year Trends in Obstetric Hemorrhage, Hemostatic Management, and Blood Product Utilization at a Tertiary Maternity Center in Kazakhstan: A Retrospective Time-Series Study

**DOI:** 10.3390/healthcare14152408

**Published:** 2026-08-05

**Authors:** Ardak Ayazbekov, Almagul Kurmanova, Saken Khaidarov, Aigul Terlikbayeva, Damilya Salimbayeva, Madina Khalmirzaeva, Shugyla Amirtayeva, Gulnara Iskakova

**Affiliations:** 1 Scientific Center of Obstetrics, Gynecology and Perinatology, Almaty 050010, Kazakhstan; ardakayazbekov@gmail.com (A.A.); aigultrk@mail.ru (A.T.); sdamilya@mail.ru (D.S.); 2 Faculty of Medicine and Healthcare, al-Farabi Kazakh National University, Almaty 050040, Kazakhstan; kurmanova.almagul@kaznu.kz (A.K.); madinakhalmirzaeva7@gmail.com (M.K.); amirtayeva@gmail.com (S.A.); 3 Department of Molecular Biology and Medical Genetics, Asfendiyarov Kazakh National Medical University, Almaty 050004, Kazakhstan; 4 Department of Public Health, Khoja Akhmet Yassawi International Kazakh-Turkish University, Turkistan 161200, Kazakhstan

**Keywords:** postpartum hemorrhage, obstetric hemorrhage, carbetocin, tranexamic acid, blood transfusion, cesarean section, uterine compression sutures, time-series analysis, maternal health, reproductive potential, fertility preservation, Kazakhstan

## Abstract

**Background/Objectives:** Obstetric hemorrhage remains a leading cause of maternal morbidity and mortality. We examined five-year institutional trends in the recorded hemorrhage rate, cesarean delivery, uterus-preserving hemostatic procedures, uterotonic and antifibrinolytic use, and blood product consumption at a high-volume tertiary maternity center in Kazakhstan. **Methods:** This was a retrospective time-series analysis of aggregated annual and monthly records for 2018–2022 (46,554 deliveries). The outcome was hemorrhage as entered on the hemorrhage line of the institutional register against the national threshold of 500 mL after vaginal birth and 1000 mL after cesarean; measured blood loss and severity grade were not recorded, so the study describes incidence rather than severity, and blood product figures are hospital-wide adult transfusion volumes. We calculated rates per 1000 deliveries with Wilson confidence intervals, fitted Poisson regression with delivery volume as an offset to estimate incidence rate ratios (IRRs), compared recorded hemorrhage by delivery route with Fisher’s exact test, screened for seasonality and out-of-limit months with process control charts, and correlated the monthly hemorrhage rate with service-use variables before and after detrending, with false-discovery-rate correction. **Results:** The recorded hemorrhage rate fell from 11.39 to 3.62 per 1000 deliveries (rate ratio 0.318, 95% CI 0.217–0.466; 68.2% relative reduction), an average decline of 27.6% per year (IRR 0.724, 95% CI 0.665–0.788, *p* < 0.001). No reproducible seasonal pattern was found. The cesarean rate declined from 24.90% to 19.36%. Carbetocin and tranexamic acid use rose several-fold, while red cell volume per delivery fell by about 48%. Raw inverse correlations between medication use and hemorrhage were explained by the shared time trend and did not survive detrending and correction. **Conclusions:** The center recorded a substantial, statistically robust decline in recorded obstetric hemorrhage alongside expanded pharmacologic prophylaxis and reduced transfusion. Because the data are aggregate and neither placental pathology nor other patient case mix could be measured, the parallel trends cannot show that any intervention caused the fall, and a shift in documentation practice cannot be excluded. The findings support prospective monitoring and patient-level study. Because severe hemorrhage and the surgery used to control it may compromise later fertility, the reproductive outcomes of these women are a priority for further research.

## 1. Introduction

Obstetric hemorrhage is still the single largest contributor to maternal death worldwide, and most of those deaths occur in settings where the bleeding could have been controlled. In the World Health Organization (WHO) systematic analysis of maternal mortality, hemorrhage accounted for roughly a quarter of deaths, more than any other single cause [1,2]. Postpartum hemorrhage (PPH) is also the component clinicians can most plausibly prevent because a large share of cases follow a fairly predictable course from uterine atony to escalating blood loss [3,4]. Atony accounts for most events in population series; abnormal placentation, retained tissue, and genital tract trauma account for the greater part of the remainder [5,6].

The toolkit for prevention and treatment has changed a great deal over the past two decades. Active management of the third stage of labor and prophylactic uterotonics became standard practice, and the range of agents widened once heat-stable carbetocin proved non-inferior to oxytocin for preventing bleeding after vaginal birth [7,8,9]. Early tranexamic acid reduced death from bleeding in the WOMAN trial and is now recommended within three hours of PPH onset [10]. When drugs are not enough, uterus-preserving surgery such as the B-Lynch compression suture and O’Leary uterine artery ligation offers an alternative to hysterectomy [11,12]. Current FIGO and WHO guidance goes further than any single agent and frames prevention and treatment as a bundle: risk assessment on admission, a prophylactic uterotonic at every birth, quantitative rather than visual estimation of blood loss, early tranexamic acid, and a written escalation pathway with a defined massive-transfusion step [13,14]. National guidelines take much the same position [15,16], and the mechanical and surgical steps sit at the end of that pathway on evidence thinner than the pharmacologic steps above them [17]. Structured hemorrhage bundles that combine readiness, recognition, and a staged response have been linked to lower blood product use and fewer severe outcomes [18,19].

How these elements come together in day-to-day practice varies between hospitals and between health systems. Several high-income registries have reported that PPH rates rose rather than fell over the same period, driven largely by atonic hemorrhage, which makes local trend data worth examining rather than assuming [20,21,22]. Reports from Central Asia are scarce, and Kazakhstan in particular has published little on institutional hemorrhage trends, despite a national reorganization of perinatal care around regional referral centers. Scarcity of publications is not the only reason to look. Kazakhstan, like other upper-middle-income countries, has been adopting the pharmacologic and organizational components of modern hemorrhage care within a single decade rather than across the thirty years over which they accumulated in high-income systems and adopting them unevenly, as procurement and training reach individual hospitals at different times. Regionalization concentrates the sickest women in a small number of tertiary centers, so what a national indicator averages away is visible at the level of one unit. Where no national PPH registry or audit exists, institutional series of this kind are the only available description of how that transition is going, and they are the raw material from which prospective monitoring is usually built.

One determinant of severe hemorrhage sits only partly within obstetric practice. Cesarean rates have risen across most regions over two decades [23,24] and with them the placenta accreta spectrum (PAS), which takes a disproportionate share of massive transfusion, peripartum hysterectomy, and uterus-preserving surgery. A recent five-year hospital series of placenta previa with abnormal adhesion shows how heavily this small group weighs on institutional blood use and surgical workload [25]. The mechanism is vascular: the placental bed is supplied by low-resistance vessels of substantial caliber, and morphometric work on placental vasculature shows how much flow the uteroplacental circulation carries at term [26], which is why failure of the myometrium to close that bed after delivery empties a woman quickly. How many such placentas a unit receives in a given year is therefore part of any institutional hemorrhage trend, whether or not it can be measured.

We therefore looked back over five years of records at a high-volume tertiary maternity center in Kazakhstan. The aim was descriptive but specific: to quantify year-on-year and month-on-month changes in the recorded obstetric hemorrhage rate; to see how cesarean delivery, uterus-preserving hemostatic procedures, uterotonic and antifibrinolytic use, and blood product consumption moved over the same window; and to test whether shifts in medication use tracked the fall in hemorrhage once the shared downward trend over time was accounted for. Because the available data are aggregate monthly and annual counts rather than individual records, we treated the study as an ecological description and were deliberately cautious about causal language.

## 2. Materials and Methods

### 2.1. Study Design and Setting

This was a retrospective, single-center time-series analysis of aggregated institutional records covering January 2018 through December 2022. The study was carried out at Regional Perinatal Center No. 3 in Turkistan, Turkistan Region, Kazakhstan, a tertiary (level III) maternity hospital that manages both routine and high-risk deliveries and acts as the referral point for obstetric emergencies across the region. Reporting follows the STROBE recommendations for observational studies where applicable [27].

### 2.2. Data Sources and Variables

Data were drawn from the hospital’s routine annual and monthly summary registers. For each month, we extracted total deliveries, cesarean sections, recorded hemorrhage events, hemorrhage by delivery route (after vaginal birth or after cesarean), B-Lynch/O’Leary hemostatic procedures with their route split, carbetocin (Pabal; Ferring Pharmaceuticals, Saint-Prex, Switzerland) doses, tranexamic acid (TXA) doses, transfused red blood cell (RBC) volume, and fresh frozen plasma (FFP) volume. The RBC and FFP figures come from the transfusion service line of the register, which records volumes issued to adult inpatients across the hospital and not to obstetric patients alone; the register holds no indication for individual transfusions. Complete monthly data were available for 2019–2022. For 2018, only annual totals were recorded for deliveries, cesarean sections, hemorrhage, carbetocin, RBC, and FFP, so 2018 contributed to the annual comparisons but not to the monthly models.

### 2.3. Data Reconciliation and Quality Control

Before analysis, we reconciled several internal inconsistencies in the source registers and recorded a handling rule for each so that the analysis can be reproduced (Table 1). Where an annual total exactly equaled the sum of the non-blank months, blank cells were read as “none recorded” and set to zero but flagged. Two conflicts touched counts that mattered for the main results: the 2020 hemorrhage total summed to 58 across months and route subtypes but appeared as 54 in the five-year summary, and the 2021 procedure rows summed inconsistently. The primary analysis used the internally reproducible monthly and subtype totals, and a sensitivity analysis repeated the main trend using the alternative 2020 value.

The discrepancies fall into two kinds, and it matters which. Most are arithmetic: a summary cell that does not equal the sum of the months feeding it, as in the 2022 cesarean total (2031 against 2030) and the 2021 procedure row (324 against 319). These are consistent with transcription at the point where monthly returns are carried into an annual summary sheet by hand, and they are small, non-systematic, and equally likely in either direction. The 2020 hemorrhage conflict (58 against 54) is of the same kind, since the monthly figures and the route subtypes agree with each other and only the summary sheet differs. The second kind is the blank cell, which carries no information about whether nothing happened or nothing was written down; in each instance, the annual total matched the sum of the non-blank months exactly, which points to a reporting convention of leaving zero months empty rather than to missing data. We found no discrepancy that suggested a change in case definition or in what was being counted. The registry is best read as an arithmetically imperfect but definitionally stable record, and the sensitivity analysis in Section 3.9 shows that the imperfections do not reach the main conclusion.

### 2.4. Definitions

“Recorded hemorrhage” is the institutional count of hemorrhage events as entered in the register by the attending team. The clinical criterion in use at the center throughout the study window is the one set out in the national clinical protocol for obstetric hemorrhage: blood loss of at least 500 mL after vaginal birth and at least 1000 mL after cesarean, estimated at the bedside and entered in the delivery or operative note. These thresholds correspond to the WHO and ACOG definitions in force over the same period [3,15]. Every event meeting the threshold was entered on the hemorrhage line whatever its severity, so the count is not restricted to severe or massive events. What the register does not hold is the estimated volume for each case, a severity grade, an etiologic category, or a field for massive transfusion, hysterectomy, or intensive care admission. The outcome is therefore a measure of incidence. It cannot distinguish a 600 mL bleed controlled with one uterotonic from a 2000 mL bleed that ended in the operating theatre, and any change in the severity mix across the five years is invisible to us. The register template, the reporting form, and the diagnostic threshold were unchanged between 2018 and 2022; no revision of institutional coding rules or of the reporting system is documented in the source registers or known to the clinical team. That is not the same as an audit of documentation practice, and Section 4 treats gradual drift in how consistently borderline cases were entered as a competing explanation for part of the observed decline. Route categories (after vaginal delivery and after cesarean) are reproduced as recorded.

Hemostatic procedures are counted per intervention performed rather than per woman treated, and the register keeps the B-Lynch compression suture and O’Leary uterine artery ligation on a single line. A woman who received both techniques at one operation therefore contributes two entries. The line also includes sutures and ligations placed during cesarean for intraoperative uterine hypotonia or for anticipated bleeding in women with risk factors, where estimated loss stayed below the diagnostic threshold and no hemorrhage was coded. Procedures and coded hemorrhage events are consequently two overlapping but non-nested counts drawn from different parts of the register, and the procedure total is not a subset of the hemorrhage total. Section 4 returns to this point. Carbetocin and tranexamic acid counts represent doses or units dispensed and cannot be tied to individual patients. RBC and FFP volumes are institution-wide adult totals and may include indications other than obstetric hemorrhage.

### 2.5. Statistical Analysis

The analysis answers four questions in sequence, and the methods below follow that order. Did the rate change over five years, and by how much? This was investigated using Poisson regression with delivery volume as an offset, reported as incidence rate ratios. Is the change a genuine trend or a recurring seasonal cycle? We determined this through a likelihood-ratio test and a Kruskal–Wallis test on calendar month. Were any individual months unusual enough to merit review? Here, we utilized a process control chart with limits that adjust for each month’s delivery volume. Did service-use variables move with the hemorrhage rate for reasons other than the shared passage of time? Spearman correlations before and after detrending, corrected for multiple testing, were used. A sensitivity analysis then repeats the first question under the alternative 2020 count. A reader who follows only the confidence intervals in Section 3 will not miss the substance. We summarized counts as rates per 1000 deliveries and cesarean delivery as a percentage, with Wilson 95% confidence intervals (CIs) for annual proportions. Heterogeneity across years was tested with the chi-square test. Time trends were modeled with Poisson regression using the natural log of delivery volume as an offset, giving incidence rate ratios (IRRs); calendar year was the predictor for the annual model and consecutive month for the monthly model. Overdispersion was checked with the Pearson chi-square/df statistic. Recorded hemorrhage by delivery route was compared with Fisher’s exact test and rate ratios. Seasonality was assessed two ways: a likelihood-ratio test comparing the trend model with and without calendar month and a Kruskal–Wallis test across months. We built a statistical process control (SPC) *p*-chart with a center line equal to the pooled 2019–2022 rate and 3σ limits that varied with each month’s denominator. Associations between the monthly hemorrhage rate and service-use variables were examined with Spearman correlations, corrected for multiple comparisons with the Benjamini–Hochberg false discovery rate (FDR) [28] and then recomputed after detrending (rank transformation with residualization on calendar time) to separate genuine co-movement from shared secular trend. Tests were two-sided with significance at *p* < 0.05, and we emphasized effect sizes and CIs over *p*-values.

The trend was fitted at two time scales because each answers a question the other cannot. The annual model is the only one that can include 2018, the year with the highest rate and with annual totals alone, so it carries the full five-year contrast; resting on five observations, it is fragile and can be moved by a single year. The monthly model gives up 2018 but gains 48 observations, which is what makes the seasonality test, the process control chart, and the detrended correlation analysis possible and gives a usable estimate of dispersion. Neither is a robustness check on the other in a formal sense, since they use overlapping data; we report both because they were built on different denominators and different time units, and their agreement (annualized IRR 0.724 versus 0.695) makes a modeling artifact less likely.

Analyses were performed in Python 3.11 (Python Software Foundation, Wilmington, DE, USA), using the open-source libraries statsmodels 0.14 and SciPy 1.11 for the statistical models and Matplotlib 3.11 for the figures.

### 2.6. Ethics

The study was conducted in accordance with the Declaration of Helsinki. The research program within which this analysis was carried out was reviewed and approved by the Local Bioethics Commission of the “Academy of Preventive Medicine” Public Association, Almaty, Kazakhstan (accredited by the Forum of Ethics Committees of Kazakhstan, certificate No. XIII-R, valid 14 December 2023 to 14 December 2026), at its meeting held on 21 May 2025 (protocol code № XIII-R—authors to insert, approval date 21 May 2025). Approval was granted after the period covered by the data because the analysis was planned within a research program launched in 2025 and used only aggregated, de-identified records already held by the institution; no patient was contacted, and no individual record was retrieved. Because the analysis used aggregated, de-identified institutional records containing no individually identifiable information, the requirement for individual informed consent was waived.

## 3. Results

### 3.1. Overview

Over the five years, the center recorded 46,554 deliveries and 288 hemorrhage events, an overall rate of 6.19 per 1000 deliveries. Annual delivery volume grew from 7373 in 2018 to a peak of 11,393 in 2021 and then eased slightly to 10,488 in 2022.

### 3.2. Annual Hemorrhage Trend

**Figure 1:**
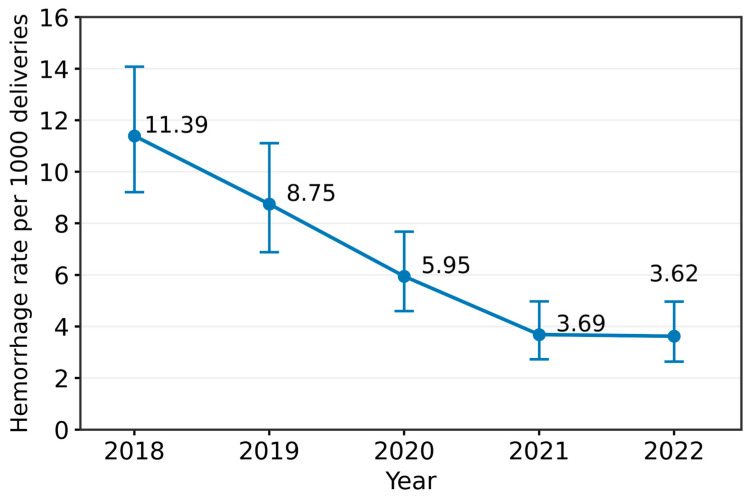
Annual recorded obstetric hemorrhage rate per 1000 deliveries with Wilson 95% confidence intervals, 2018–2022.

The recorded hemorrhage rate fell year on year (Table 2, Figure 1), and the difference across years was large and unlikely to be chance (χ^2^ = 63.44, df = 4, *p* < 0.001). Comparing the endpoints, the 2022 rate was about a third of the 2018 rate (rate ratio 0.318, 95% CI 0.217–0.466), an absolute reduction of 7.77 hemorrhages per 1000 deliveries (95% CI 5.09–10.45). The Poisson model put the average decline at 27.6% per calendar year (IRR 0.724, 95% CI 0.665–0.788, *p* < 0.001), with no sign of problematic overdispersion (dispersion 0.70).

### 3.3. Monthly Pattern, Seasonality, and Process Control

**Figure 2:**
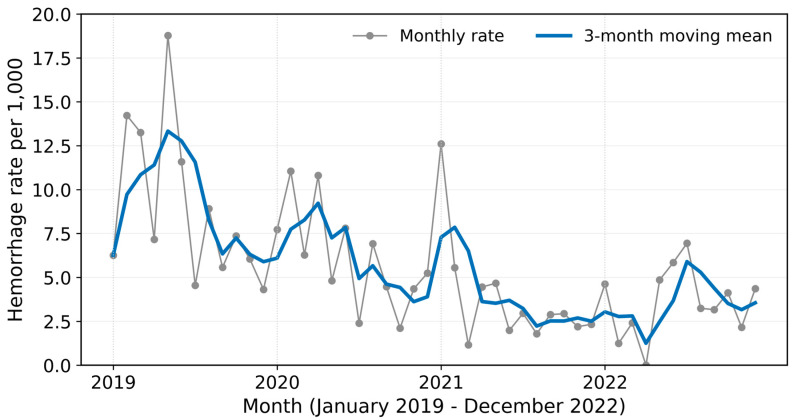
Monthly recorded hemorrhage rate and three-month moving mean, January 2019–December 2022.

**Figure 3:**
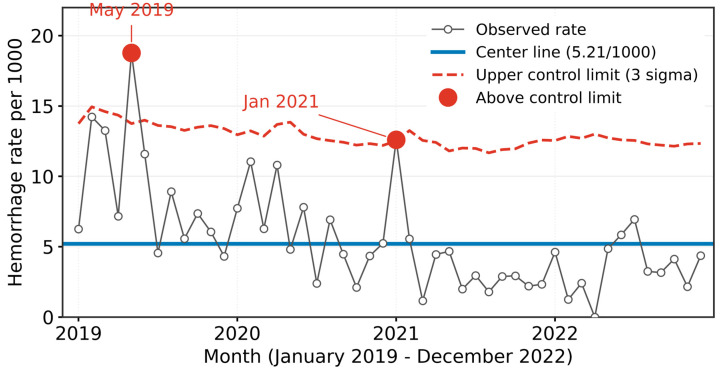
Statistical process control *p*-chart. The center line is the pooled 2019–2022 rate (5.21 per 1000); upper 3σ control limits vary with each month’s delivery volume. Two months exceeded the limit.

The monthly series told the same story with more texture (Table 3, Figure 2). Fitted month by month, the rate fell by about 3% per month (IRR 0.9702, 95% CI 0.9601–0.9804, *p* < 0.001), which annualizes to 0.695 (95% CI 0.613–0.788); the dispersion statistic of 1.14 was consistent with the Poisson assumption. Adding the calendar month to the trend model did not improve fit (likelihood-ratio χ^2^ = 9.00, df = 11, *p* = 0.622), and a Kruskal–Wallis test was likewise negative (H = 8.22, *p* = 0.693), so we found no reproducible seasonal cycle. Two months crossed the upper 3σ control limit on the *p*-chart for May 2019 and January 2021 (Table 4, Figure 3). Both were isolated spikes rather than part of a recurring pattern, and they are the kind of signal that warrants a focused chart review.

### 3.4. Cesarean Delivery

**Figure 4:**
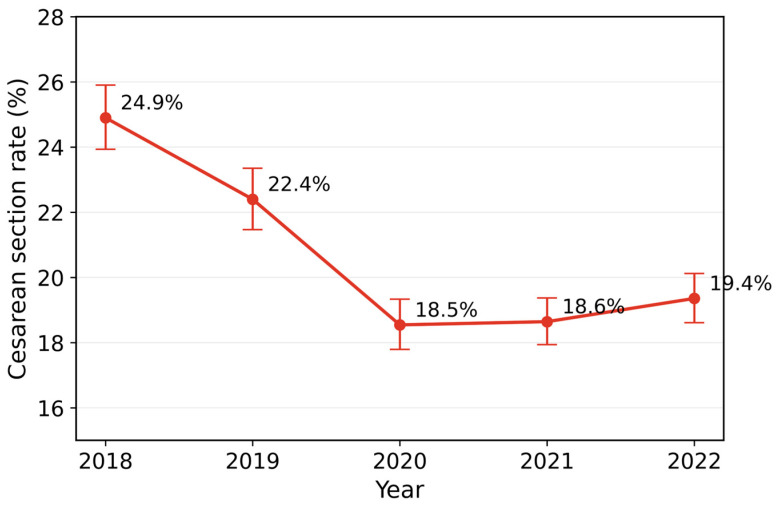
Annual cesarean section rate with Wilson 95% confidence intervals, using the reconciled 2022 count of 2030.

The cesarean rate also fell over the window, from 24.90% in 2018 to 19.36% in 2022 (Table 2, Figure 4), again a difference unlikely to be chance (χ^2^ = 160.06, df = 4, *p* < 0.001). The path was not a straight line: the rate bottomed out at 18.54% in 2020 and then edged up over the next two years. The endpoint rate ratio was 0.777 (95% CI 0.735–0.822). Both the starting and ending figures are above the 10–15% band that the WHO statement associates with population-level benefit [24].

### 3.5. Hemorrhage by Delivery Route

Split by route, the recorded rate over 2019–2022 was 6.06 per 1000 vaginal deliveries and 1.70 per 1000 cesarean deliveries (Table 5), a rate ratio of 3.57 (95% CI 2.03–6.25; Fisher’s exact *p* < 0.001). This contrast should be read with care. The categories are institutional labels applied to aggregate counts, so the gap could reflect how events were coded, differences in prophylaxis, or reporting practice as much as any real difference in risk between routes. We do not interpret it as an adjusted clinical comparison.

### 3.6. Uterus-Preserving Hemostatic Procedures

A total of 1042 B-Lynch/O’Leary procedures were recorded over 2019–2022, of which 982 (94.2%) followed cesarean delivery (Table 6). Normalized to delivery volume, the procedure rate stayed broadly flat (IRR per year 0.985, 95% CI 0.931–1.042, *p* = 0.600), moving between about 24 and 29 per 1000 deliveries without a clear trend even as the hemorrhage rate fell. The procedure count over these four years is five times the coded hemorrhage count for the same period (1042 versus 204 events), and the two series do not move together. This follows from how the register is constructed rather than from a contradiction in the data: procedures are counted per intervention on a line shared by two techniques, and they include applications at cesarean below the diagnostic threshold for hemorrhage (Section 2.4). Expressed against surgical rather than delivery volume, the 982 post-cesarean procedures correspond to 12.8% of the 7653 cesareans performed over the same period.

### 3.7. Medication and Transfusion Utilization

**Figure 5:**
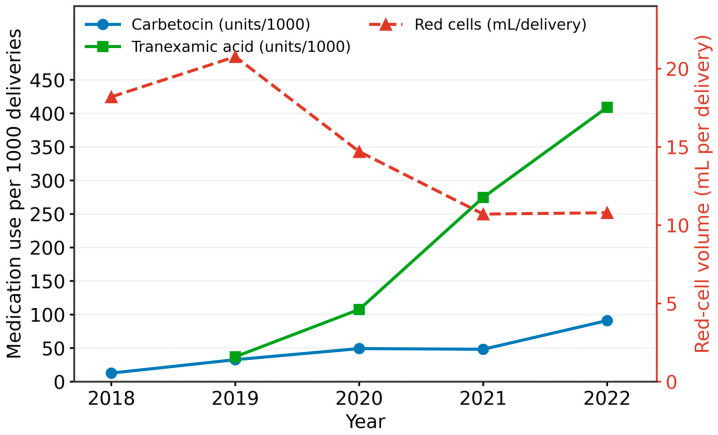
Carbetocin and tranexamic acid use per 1000 deliveries (left axis) and transfused red cell volume per delivery (right axis), 2018–2022. Tranexamic acid was not recorded in 2018.

Recorded use of both pharmacologic agents rose sharply over the five years, carbetocin roughly sevenfold and tranexamic acid roughly elevenfold when normalized to delivery volume (Table 7, Figure 5). Transfused red cell volume per delivery moved the other way, falling by about 48% between 2019 and 2022. FFP volume per delivery remained low throughout; the 2022 figure covers only January–November and is flagged as partial. Red cell and plasma volumes are hospital-wide adult totals rather than obstetric consumption, and the denominator used here (deliveries) normalizes them to obstetric activity only for comparability across years.

### 3.8. Correlation Analysis

At the monthly level, higher carbetocin and tranexamic acid use lined up with lower hemorrhage rates and higher red cell use with higher hemorrhage rates (Table 8). These raw correlations were moderate to strong and, for the medications and red cells, statistically significant before adjustment. They did not survive scrutiny. The series carry strong secular trends, with hemorrhage and transfusion falling while medication use climbed, so much of the apparent relationship is shared movement over time. After detrending and FDR correction, none of the associations remained significant. The correlation analysis therefore does not show that any medication reduced hemorrhage; it mainly shows that several things changed together across the same five years.

### 3.9. Sensitivity Analysis

Substituting the conflicting 2020 summary count (54 instead of 58) left the trend essentially unchanged, and the monthly model gave a consistent annualized estimate (Table 9). The direction, size, and significance of the decline held under both assumptions.

## 4. Discussion

Across five years and roughly 46,000 deliveries, this tertiary center recorded a steady, sizable fall in coded obstetric hemorrhage, down about two-thirds from 2018 to 2022, with an average decline near 28% a year that held up under sensitivity analysis. Over the same window the cesarean rate came down, carbetocin and tranexamic acid use rose several-fold, and red cell transfusion per delivery roughly halved. Uterus-preserving procedures stayed flat and were almost entirely a post-cesarean phenomenon. What we did not find matters as much as what we did: no seasonal cycle and no medication–hemorrhage association that survived once the shared time trend was removed.

The outcome itself deserves attention before any of that is interpreted. The numerator is an event coded by the treating team against a volume threshold estimated at the bedside, with no recorded volume, severity grade, or cause [3,15]. Two things follow. First, this is a study of incidence and says nothing about severity; reducing the number of coded events and the proportion that become life threatening are different achievements, measured differently, and severe PPH has risen in settings where overall rates were stable [21]. The halving of red cell volume per delivery is our only signal of severity, but it is weak because the transfusion line is hospital-wide. Second, an incidence measure resting on visual estimation is sensitive to documentation. Visual estimation under-reads blood loss, and units that adopt quantitative measurement usually see their recorded rate rise rather than fall [13]. Neither the threshold, the register form, nor the reporting system changed at this center over the study window, as far as the registers and the clinical team’s records indicate. That is worth stating, but it is not an audit, and a gradual change in how consistently borderline cases were entered would look very much like part of the decline we report. We regard this as the most serious competing explanation for our main finding, and aggregate data cannot settle it.

A related gap concerns what the unit itself was doing. Over these five years, the center reorganized as part of the national regionalization of perinatal care, carbetocin and tranexamic acid were added to routine stock, and national clinical protocols were revised in line with international guidance. We cannot put dates on any of these. The registers hold no record of when a protocol was adopted, when simulation or skills training was delivered, whether a quality-improvement cycle for hemorrhage ran, or when the obstetric and midwifery staff turned over. Each of these plausibly shifts a hemorrhage rate, and one of them, a formal hemorrhage bundle introduced at a known date, would move this study from a correlational description to an interrupted time series with a testable break point [18,19]. We flag them as unmeasured contributors to the trend rather than as a list of things that did not happen. Reconstructing the implementation calendar from administrative orders and training records is a realistic next step for the center and would be worth more than another year of aggregate counts.

The procedure counts show the same problem from another angle. The register holds 1042 B-Lynch/O’Leary procedures for 2019–2022 against 204 coded hemorrhage events. As a subset, that is impossible, and it is not one. Procedures are counted per intervention on a row shared by compression suture and uterine artery ligation, so a woman treated with both appears twice, and the row includes sutures and ligations placed at cesarean for intraoperative hypotonia or anticipated bleeding where estimated loss stayed under the threshold and no hemorrhage was coded. Set against surgical volume, the figure is 982 procedures across 7653 cesareans, about 1 operation in 8. That is high, and it fits a referral unit that has moved toward early surgical hemostasis at cesarean rather than waiting for a threshold to be crossed, in a field where the surgical evidence base remains thin [17]. The procedure line therefore tracks operative practice, while the hemorrhage line tracks events that crossed a volume threshold, and over these five years, the two moved independently. Only a patient-level extract can reconcile them.

We have no patient covariates, and the gap that concerns us most is the placental one. Placenta previa and PAS take a disproportionate share of massive hemorrhage, transfusion, hysterectomy, and uterus-preserving surgery, and their incidence at any one hospital is not stable; it follows the cesarean history of the population served and the referral pattern of the region [23,25]. A center receiving fewer accreta cases in 2022 than in 2018 would show a lower hemorrhage rate, fewer compression sutures, and a smaller transfusion volume with no change whatever in practice. The same holds for maternal age, parity, previous cesarean, induction of labor, multiple pregnancy, hypertensive disorders, anemia, and fetal macrosomia, none of which the register carries; nor does it carry hysterectomy or intensive care admission, so the most severe end of the spectrum is absent altogether. The falling cesarean rate is the only case-mix variable we can observe and a poor proxy for the rest. Whatever contribution shifting case mix made to the trend, we cannot quantify it, and our confidence intervals take no account of it.

With those limits stated, the direction of the trend is still worth reporting. It runs against much of what large high-income registries have found, where PPH rates rose over comparable periods, mostly through atonic hemorrhage [20,21,22]. A local decline is not implausible: expanding prophylaxis, protocolized response, and referral patterns can all push that way, and the parallel rise in carbetocin and tranexamic acid follows the trials and guidance that reshaped PPH care over this period [8,9,10]. What we cannot do is join the two. The raw monthly correlations were inverse and significant; they vanished after detrending and FDR correction, which is what happens when two series move across the same five years for reasons that may be unrelated. Procurement lifted prescribing in steps while hemorrhage fell gradually, and an aggregate correlation cannot separate a treatment effect from a coincidence of timing. We therefore set the trends side by side and make no claim that carbetocin or tranexamic acid reduced hemorrhage at this center; testing that would need patient-level exposure data and, better still, an interrupted time series anchored to the dates on which each agent entered routine use [29].

The fall in red cell volume per delivery, to nearly half, was inversely related to medication use. Earlier and more effective control of bleeding would produce that pattern. So would a stricter transfusion threshold adopted for its own sake, a hospital-wide patient blood management program, or a change in the non-obstetric adult case mix sharing the same transfusion line [19,30]. Since the line is institution-wide and carries no indication, we cannot choose between these.

The route-specific contrast warrants caution. Read naively, a 3.6-fold higher recorded rate after vaginal birth might suggest vaginal delivery is riskier, which would invert the usual clinical understanding that cesarean carries substantial hemorrhage risk. The likelier explanation is definitional: how the hospital assigns and counts hemorrhage after vaginal delivery versus after cesarean and the possibility that post-cesarean bleeds were managed and coded under the procedure line rather than the hemorrhage line. We flag this as a data artifact to be resolved with patient-level records, not as a finding. The two out-of-limit months point the other way, toward what aggregate data can still do: process control turned a noisy series into two reviewable signals (May 2019 and January 2021) and left the rest of the variation alone. Run prospectively and risk-adjusted, that is monitoring that catches clustering while the charts are still fresh.

The study’s strengths are its length and size, five continuous years and about 46,000 deliveries from a single high-volume center, and the explicit reconciliation of the source registers, with a sensitivity analysis confirming that the main conclusion does not hinge on the disputed 2020 count. The limitations run the other way and have been set out above: an ecological design open to the ecological fallacy, so that associations between monthly aggregates need not hold for individual women; an outcome coded rather than measured, with no severity grade; no patient covariates and in particular no placental diagnoses; procedure and medication counts that are interventions and doses rather than women; blood product totals that are not obstetric-specific; blank cells interpreted under the rules in Table 1; a missing December 2022 FFP figure; and a single center in a single country.

One consequence of hemorrhage falls outside what these registers can see. Hysterectomy ends fertility outright; uterus-preserving surgery avoids that, although compression sutures and uterine artery ligation have been followed by intrauterine adhesions, menstrual disturbance, and impaired fertility in a proportion of reported series [31,32,33]; embolization raises similar questions [34]; and massive transfusion carries a risk of pituitary necrosis [35]. Longer follow-up of other uterus-preserving obstetric surgery is more reassuring, with low recurrence and satisfactory subsequent pregnancies after myomectomy at cesarean [36]. We raise the question only because the 1042 procedures recorded here represent a sizable group of women whose later menstrual function and pregnancies are unknown to us. We did not evaluate these outcomes, and our data do not speak to them.

What follows is a patient-level dataset: multivariable modeling of hemorrhage risk with placental diagnoses included, severity grading, propensity-based evaluation of carbetocin, tranexamic acid, and surgical hemostasis, and an interrupted time series tied to the dates on which protocols or medication policies changed. Correcting the registry inconsistencies documented in Table 1 would make it more reliable overall.

## 5. Conclusions

Over 2018–2022, Regional Perinatal Center No. 3 in Turkistan recorded a substantial and statistically robust decline in coded obstetric hemorrhage alongside expanded use of carbetocin and tranexamic acid and a roughly halved red-cell transfusion volume per delivery. The trends are coherent and consistent with modern hemorrhage prevention practice. They do not establish that any single intervention caused the fall. The medication–hemorrhage correlations disappeared once the common time trend was removed, placental pathology and other case-mix variables went unmeasured, and a gradual change in documentation practice cannot be excluded from aggregate records.

A falling incidence also measures only part of the harm: severity was not recorded, and the reproductive consequences for women treated for severe hemorrhage lie outside these registers entirely. Three lines of work follow: prospective, risk-adjusted monitoring at the unit level; a patient-level study that converts these institutional patterns into adjusted individual-level evidence; and a follow-up cohort of women treated for severe hemorrhage in which menstrual function, fertility, and the course of the subsequent pregnancy are recorded systematically.

## Figures and Tables

**Table 1:** Data reconciliation and quality checks applied before analysis.

Year	Issue Identified	Analytical Handling
2018	Hemorrhage count (84) available only in the five-year summary; no monthly hemorrhage or procedure data.	Included in annual analyses only.
2019	Tranexamic acid blank for January–June; annual total equals the July–December sum.	Blank months treated as none recorded.
2020	Monthly hemorrhage and route subtypes sum to 58; five-year summary reports 54.	Primary analysis uses 58; sensitivity uses 54.
2021	Procedure total row sums to 324; subtype rows and annual total sum to 319.	Procedure total reconstructed from route subtypes (319).
2021	Carbetocin blank in January and November; annual total equals the sum of other months.	Blank months treated as none recorded.
2022	Monthly cesarean sections sum to 2030 vs. reported 2031.	Analysis uses the reproducible monthly sum (2030).
2022	December FFP blank; annual total equals the January–November sum.	2022 FFP treated as an 11-month partial total and flagged.

**Table 2:** Annual deliveries, recorded hemorrhages, and cesarean sections, 2018–2022, with Wilson 95% confidence intervals. Primary reconciled counts are shown.

Year	Deliveries	Hemorrhages	Rate/1000 (95% CI)	Cesarean	CS Rate, % (95% CI)
2018	7373	84	11.39 (9.21–14.08)	1836	24.90 (23.93–25.90)
2019	7545	66	8.75 (6.88–11.11)	1690	22.40 (21.47–23.35)
2020	9755	58	5.95 (4.60–7.68)	1809	18.54 (17.79–19.33)
2021	11,393	42	3.69 (2.73–4.98)	2124	18.64 (17.94–19.37)
2022	10,488	38	3.62 (2.64–4.97)	2030	19.36 (18.61–20.12)

**Table 3:** Monthly descriptive statistics for recorded hemorrhages, 2019–2022. SD, standard deviation; IQR, interquartile range.

Year	Count, Mean ± SD	Median (IQR)	Rate/1000, Mean ± SD	Rate Range
2019	5.50 ± 2.54	4.5 (4.0–7.0)	9.00 ± 4.51	4.32–18.78
2020	4.83 ± 1.90	5.0 (3.8–6.0)	6.16 ± 2.87	2.11–11.05
2021	3.50 ± 2.61	3.0 (2.0–4.0)	3.79 ± 3.06	1.16–12.60
2022	3.17 ± 1.70	3.5 (2.0–4.0)	3.58 ± 1.95	0.00–6.94

**Table 4:** Months exceeding the upper statistical process control limit. UCL, upper control limit.

Month	Deliveries	Hemorrhages	Observed Rate/1000	UCL/1000	Signal
May 2019	639	12	18.78	13.75	Above UCL
January 2021	873	11	12.60	12.51	Above UCL

**Table 5:** Route-specific recorded hemorrhage rates, 2019–2022. Categories are reproduced from the source register.

Year	Vaginal Hem./Deliveries	Rate/1000	Post-CS Hem./CS	Rate/1000
2019	61/5855	10.42	5/1690	2.96
2020	54/7946	6.80	4/1809	2.21
2021	41/9269	4.42	1/2124	0.47
2022	35/8458	4.14	3/2030	1.48
Pooled	191/31,528	6.06	13/7653	1.70

**Table 6:** Reconciled B-Lynch/O’Leary procedure counts and rates, 2019–2022. CS, cesarean section. Procedures are counted per intervention, not per woman.

Year	Total Procedures	Per 1000	After Vaginal	After CS	Post-CS Share, %
2019	189	25.05	15	174	92.1
2020	278	28.50	21	257	92.4
2021	319	28.00	16	303	95.0
2022	256	24.41	8	248	96.9

**Table 7:** Medication and blood product utilization, 2018–2022. The /1000 columns give the recorded count per 1000 deliveries. Counts may represent units or doses rather than unique patients; RBC and FFP are hospital-wide adult volumes. * The 2022 FFP total contains January–November only. NA, not available.

Year	Carbetocin	/1000	TXA	/1000	RBC, L	RBC mL/del.	FFP, L	FFP mL/del.
2018	94	12.7	NA	NA	134.2	18.20	13.1	1.77
2019	247	32.7	280	37.1	156.7	20.76	34.3	4.55
2020	481	49.3	1050	107.6	143.4	14.70	18.8	1.93
2021	550	48.3	3130	274.7	122.0	10.71	23.4	2.05
2022	955	91.1	4290	409.0	113.2	10.79	28.4 *	2.71 *

**Table 8:** Spearman correlations between the monthly hemorrhage rate and service-use variables (2019–2022). Detrended correlations control linearly for calendar time after rank transformation. FDR q, Benjamini–Hochberg adjusted *p*-value.

Variable	*n*	Raw ρ	Raw *p*	Raw q	Detr. ρ	Detr. *p*	Detr. q
Cesarean section rate (%)	48	0.296	0.041	0.057	0.068	0.646	0.754
B-Lynch/O’Leary per 1000	48	0.050	0.737	0.737	0.075	0.614	0.754
Carbetocin per 1000	48	−0.431	0.002	0.004	0.008	0.956	0.956
Tranexamic acid per 1000	48	−0.685	<0.001	<0.001	−0.338	0.019	0.066
RBC volume (mL/delivery)	48	0.661	<0.001	<0.001	0.364	0.011	0.066
FFP volume (mL/delivery)	47	0.230	0.120	0.140	0.220	0.137	0.240
Monthly delivery volume	48	−0.623	<0.001	<0.001	−0.311	0.031	0.073

**Table 9:** Sensitivity analyses for the time trend in the recorded hemorrhage rate.

Analysis	Assumption	IRR (95% CI)	*p*-Value
Primary hemorrhage trend	2020 count = 58 (monthly and subtype total)	0.724 (0.665–0.788)	<0.001
Sensitivity hemorrhage trend	2020 count = 54 (five-year summary)	0.722 (0.663–0.786)	<0.001
Monthly trend, 2019–2022	48 monthly observations, annualized	0.695 (0.613–0.788)	<0.001

## Data Availability

The aggregated monthly dataset supporting the analysis is provided within the article (Appendix A). Further aggregated data are available from the corresponding author on reasonable request, subject to institutional approval.

## Abbreviations

ACOG, American College of Obstetricians and Gynecologists; CI, confidence interval; CS, cesarean section; FDR, false discovery rate; FFP, fresh frozen plasma; FIGO, International Federation of Gynecology and Obstetrics; IQR, interquartile range; IRR, incidence rate ratio; PAS, placenta accreta spectrum; PPH, postpartum hemorrhage; RBC, red blood cell; RR, rate ratio; SD, standard deviation; SPC, statistical process control; TXA, tranexamic acid; UCL, upper control limit; WHO, World Health Organization.

## Appendix A. Reconciled Monthly Analysis Dataset, 2019–2022

**Table A1:** Reconciled monthly dataset used for the analysis. B-L/O’L, B-Lynch/O’Leary procedures; hem., hemorrhage; vag., vaginal; Carbet., carbetocin; TXA, tranexamic acid. December 2022 FFP was missing (NA). Procedure counts are per intervention, not per woman. NA, not available.

Month	Deliv.	CS	CS%	Hem.	Hem./1000	Vag. Hem.	CS Hem.	B-L/O’L	Carbet.	TXA	RBC, L	FFP, L
January 2019	639	143	22.4	4	6.26	4	0	9	15	0	11.55	0.86
February 2019	492	126	25.6	7	14.23	6	1	11	10	0	10.84	0.84
March 2019	528	127	24.1	7	13.26	7	0	16	25	0	9.08	1.50
April 2019	558	154	27.6	4	7.17	4	0	10	20	0	13.63	2.86
May 2019	639	150	23.5	12	18.78	10	2	9	25	0	16.27	0.30
June 2019	604	124	20.5	7	11.59	7	0	9	15	0	15.78	4.20
July 2019	659	140	21.2	3	4.55	3	0	14	30	30	13.86	1.95
August 2019	673	148	22.0	6	8.92	5	1	19	20	80	12.82	4.12
September 2019	718	161	22.4	4	5.57	4	0	18	22	60	8.67	3.20
October 2019	679	148	21.8	5	7.36	4	1	19	20	40	12.42	7.23
November 2019	661	130	19.7	4	6.05	4	0	23	20	10	17.64	5.82
December 2019	695	139	20.0	3	4.32	3	0	32	25	60	14.09	1.44
January 2020	776	158	20.4	6	7.73	6	0	36	25	100	15.80	1.89
February 2020	724	144	19.9	8	11.05	6	2	19	25	0	10.52	1.04
March 2020	796	170	21.4	5	6.28	5	0	24	40	50	16.10	1.86
April 2020	648	115	17.7	7	10.80	6	1	19	45	60	11.49	2.09
May 2020	624	100	16.0	3	4.81	3	0	17	40	20	5.28	0.00
June 2020	769	113	14.7	6	7.80	6	0	21	70	70	14.19	0.00
July 2020	835	147	17.6	2	2.40	2	0	35	60	80	12.55	3.00
August 2020	867	154	17.8	6	6.92	5	1	19	40	130	13.98	2.26
September 2020	895	186	20.8	4	4.47	4	0	25	49	100	13.89	3.23
October 2020	948	162	17.1	2	2.11	2	0	16	40	110	4.96	0.00
November 2020	920	174	18.9	4	4.35	4	0	23	45	140	11.69	0.49
December 2020	953	186	19.5	5	5.25	5	0	24	2	190	12.91	2.97
January 2021	873	160	18.3	11	12.60	10	1	26	0	70	14.98	4.42
February 2021	720	131	18.2	4	5.56	4	0	26	35	20	10.31	2.16
March 2021	864	157	18.2	1	1.16	1	0	24	30	250	7.74	0.50
April 2021	898	172	19.2	4	4.45	4	0	22	30	200	9.69	0.00
May 2021	1071	192	17.9	5	4.67	5	0	28	35	250	14.23	4.65
June 2021	1007	172	17.1	2	1.99	2	0	42	50	280	14.09	2.91
July 2021	1015	163	16.1	3	2.96	3	0	24	70	280	10.04	1.74
August 2021	1115	208	18.7	2	1.79	2	0	33	95	380	9.42	1.71
September 2021	1040	212	20.4	3	2.88	3	0	19	55	340	8.94	1.73
October 2021	1022	181	17.7	3	2.94	3	0	19	70	350	4.93	1.25
November 2021	910	198	21.8	2	2.20	2	0	24	0	390	8.67	0.59
December 2021	858	178	20.7	2	2.33	2	0	32	80	320	8.94	1.73
January 2022	865	176	20.3	4	4.62	4	0	23	80	560	11.69	3.37
February 2022	800	158	19.8	1	1.25	1	0	25	60	390	12.49	3.44
March 2022	829	157	18.9	2	2.41	2	0	20	60	550	8.94	1.16
April 2022	767	144	18.8	0	0.00	0	0	10	100	200	11.39	3.03
May 2022	823	139	16.9	4	4.86	4	0	22	80	400	13.18	3.05
June 2022	855	175	20.5	5	5.85	5	0	27	85	285	5.98	1.22
July 2022	864	138	16.0	6	6.94	5	1	25	80	285	12.21	3.03
August 2022	925	185	20.0	3	3.24	3	0	20	70	320	9.51	4.38
September 2022	948	158	16.7	3	3.16	3	0	23	70	320	7.43	3.08
October 2022	969	175	18.1	4	4.13	3	1	22	50	260	7.37	1.66
November 2022	926	207	22.4	2	2.16	2	0	11	90	340	5.40	1.02
December 2022	917	218	23.8	4	4.36	3	1	28	130	380	7.61	NA
